# Isoform-Selective NFAT Inhibitor: Potential Usefulness and Development

**DOI:** 10.3390/ijms22052725

**Published:** 2021-03-08

**Authors:** Noriko Kitamura, Osamu Kaminuma

**Affiliations:** 1 Laboratory of Allergy and Immunology, Tokyo Metropolitan Institute of Medical Science, Tokyo 156-8506, Japan; kitamura-nr@igakuken.or.jp; 2 Department of Disease Model, Research Institute of Radiation Biology and Medicine, Hiroshima University, Hiroshima 734-8553, Japan

**Keywords:** aortic smooth muscle cell, calcineurin-binding region, cytokine, immunoprecipitation, immunosuppressant, knockout mouse, molecular operating environment, nuclear localization signal

## Abstract

Nuclear factor of activated T cells (NFAT), which is the pharmacological target of immunosuppressants cyclosporine and tacrolimus, has been shown to play an important role not only in T cells (immune system), from which their name is derived, but also in many biological events. Therefore, functional and/or structural abnormalities of NFAT are linked to the pathogenesis of diseases in various organs. The NFAT protein family consists of five isoforms, and each isoform performs diverse functions and has unique expression patterns in the target tissues. This diversity has made it difficult to obtain ideal pharmacological output for immunosuppressants that inhibit the activity of almost all NFAT family members, causing serious and wide-ranging side effects. Moreover, it remains unclear whether isoform-selective NFAT regulation can be achieved by targeting the structural differences among NFAT isoforms and whether this strategy can lead to the development of better drugs than the existing ones. This review summarizes the role of the NFAT family members in biological events, including the development of various diseases, as well as the usefulness of and problems associated with NFAT-targeting therapies, including those dependent on current immunosuppressants. Finally, we propose a novel therapeutic strategy based on the molecular mechanisms that enable selective regulation of specific NFAT isoforms.

## 1. Introduction

Nuclear factor of activated T cells (NFAT) was identified as a transcriptional regulator of interleukin-2 (IL-2) in activated T cells by Crabtree’s group in 1988 [1]. In 1993, Rao’s group reported that the pharmacological actions of both cyclosporine and FK506 (tacrolimus), which are used to attenuate transplant rejection, were achieved via inhibition of NFAT activity in T cells [2]. Thus, much attention has been paid to the importance of NFAT in T cell function and differentiation [3,4]. However, NFAT family members are involved in the development, differentiation, and function of various tissues, such as the nervous, cardiovascular, and bone metabolism systems, as well as the immune system [5,6,7].

**Figure 1:**
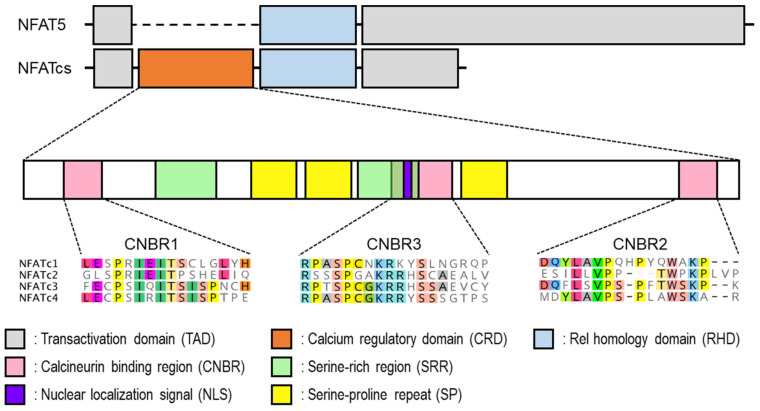
Differences in the structure, functional domains, and amino acid sequences of nuclear factor of activated T cell (NFAT) family members.

The NFAT gene family consists of five isoforms (NFATc1-c4, NFAT5), commonly involving a Rel-homology domain (RHD) that is responsible for DNA binding. NFAT5 is always distributed in both the cytoplasm and nucleus, and its activity is mainly regulated by osmotic pressure. Under high osmotic stress, NFAT5 participates in maintaining cellular functions and homeostasis [8,9]. NFAT5 also plays a role, even under isotonic conditions, in adjusting the intracellular environment suitable for cell proliferation [9]. On the other hand, NFATc1-c4 (NFATcs) are normally present in the cytoplasm, and their activities are regulated by the Ca^2+^/calmodulin-dependent serine/threonine phosphatase, calcineurin (CN) [10,11]. Upon activation with an increase in intracellular Ca^2+^ concentration, CN directly binds to the calcium regulatory domain (CRD) of NFATcs located at the *N*-terminal region of RHD and dephosphorylates its multiple phosphoserine residues (Figure 1). The subsequent conformational changes and exposure of the nuclear localization signal (NLS) enable NFATcs to translocate into the nucleus and promote the transcription of target genes by binding to the corresponding sequence of their transcription modulatory region [12,13,14]. Since the NFAT family is involved in many biological events, its functional and structural abnormalities often cause various diseases. Therefore, many researchers have proposed new means to regulate NFAT, but it has not yet reached clinical application. To overcome the present situation, this review describes the significance and feasibility of isoform-selective regulation of NFATcs, with the introduction of previous studies including our recent findings.

## 2. Functional Diversity of NFAT Family Members

NFAT is expressed in various cells and tissues and participates in the transcription of many genes, not just the initially discovered IL-2. Moreover, individual NFAT isoforms do not show the same expression patterns or functional characteristics in each cell type and/or organ (Supplementary Figures S1–S5). Based on the history of their discovery, T cells have been regarded as the principal sites of NFAT function, although NFATc4 is rarely expressed in T cells [15]. NFATc3 is more dominantly expressed than NFATc1 or NFATc2 in the thymus, while NFATc1 is highly expressed in muscle cells [12,16]. Studies using genetically modified mice have also revealed multiple functions of NFAT in the developmental processes of various organs (Table 1). NFATc1 is involved in the development of the endocardium, valves, and septum, while NFATc3 and NFATc4 control the expression of genes that regulate vascular patterning and vascularization. Consistently, mice deficient for NFATc1 or NFATc3/NFATc4 exhibit embryonic lethality due to organ hypoplasia [5,17,18]. NFAT5 knockout mice are mostly embryonic lethal caused by renal atrophy or decreased cardiac function [19,20]. NFATc2-deficient mice are born and grow normally but exhibit enhanced immune responsiveness, including splenomegaly [21,22]. NFATc4-deficiency has little effect on growth but does affect abnormal spatial memory formation in mice [23]. Conversely, NFATc4 transgenic mice develop cardiac hypertrophy and heart failure [24].

Furthermore, NFAT plays important roles in modulating cell survival, differentiation, and proliferation by regulating the expression of various genes involved in cell cycle and apoptosis, such as CDK4, cyclin, c-Myc, and FasL, as well as many cytokines and growth factors [12,25,26,27]. Through the induction of neural activity-regulating molecules, NFAT participates in the development and maintenance of the neural system [6,23]. NFATc1 plays a key role in bone metabolism, especially as a major regulator of osteoclasts [28,29].

Since NFAT regulates many important genes as described above, abnormal NFAT function often leads to the development and progression of various diseases. Constitutive activation of NFATc1 facilitates cell cycle progression and induces transformation, resulting in tumor development [30,31]. Moreover, NFATc1 participates in cancer infiltration and metastasis by inducing angiogenesis and lymphangiogenesis through the up-regulation of cyclooxygenase 2, vascular endothelial growth factor (VEGF), and CXC chemokine receptor 7 expression [32,33,34]. Clinically, NFATc1 is overexpressed in various types of tumors, such as pancreatic cancer, colorectal cancer, various leukemias, and lymphomas [31,35,36,37,38,39]. NFATc1 overexpression has been reported to be associated with poor prognosis in some cancers [40]. On the other hand, NFATc2 is implicated in an inhibitory effect on tumorigenesis, including induction of cell cycle arrest and apoptosis; thus, spontaneous lymphoma development was observed in NFATc2-deficient mice [41,42]. Nevertheless, enhanced expression of NFATc2 has been reported in patients with metastatic cancers, invasive cancers, and highly malignant cancers [34,43,44,45], suggesting that functional changes in NFATc2 may induce angiogenic dysregulation in cancer cells. The participation of NFATc3 and NFATc4 in tumorigenesis has been suggested [46,47], whereas their inhibitory effects on metastasis and infiltration in estrogen receptor-positive breast cancer [48], as well as that on lymphoma formation caused by retroviral infection [49], were also reported. Each NFAT isoform appears to play different roles depending on the type and progression of cancer.

Among the neurodegenerative disorders, a strong causal relationship has been suggested between Alzheimer’s disease (AD) and abnormal activity of NFATcs. Analysis of the human postmortem brain demonstrated that NFATc2 and NFATc4 accumulate in the hippocampus in subjects with mild cognitive impairment, severe dementia, and AD, respectively [50]. The levels of amyloid β, which is known to increase in the brain according to the severity of dementia, was correlated with that of nuclear NFATc4 [50]. Introduction of constitutively active NFATc4 into nerve cells reproduced amyloid β-induced morphological neurodegenerative changes in vitro [51]. In AD, dysregulation of intracellular calcium, which affects the enzymatic activity of CN, was observed in nerve cells [50]. The involvement of the CN/NFAT-dependent pathway in α-synuclein-induced degeneration of midbrain dopaminergic neurons in Parkinson’s disease has been suggested [52].

NFATcs are also key molecules that induce immune tolerance because they regulate the expression of Foxp3 and CTLA4, which are essential for the development and function of regulatory T cells [53,54,55,56,57]. Furthermore, NFATcs control activation-induced cell death through FasL upregulation [53,58,59]. Accordingly, anomalous NFAT activity results in the development of autoimmune and inflammatory diseases. NFAT is involved in many cellular processes typically observed in the synovium of patients with rheumatoid arthritis (RA), including activation of inflammatory cells, production of various cytokines, VEGF-mediated pathologic angiogenesis, and osteoclast formation [60]. Mice lacking leucine-rich repeat kinase 2, which is a negative regulator of NFAT and a major susceptibility gene in Crohn’s disease, displayed exacerbated experimental colitis accompanied by the activation of NFATc2 [61]. The severity of experimental allergic encephalomyelitis was diminished in mice deficient in both NFATc1 and NFATc2 [62], suggesting that NFAT also contributes to the development of multiple sclerosis. Through the regulation of macrophage activity [63] and high osmolarity-dependent pathogenic Th17 cell induction [64], NFAT5 may participate in the pathogenesis of RA and other autoimmune diseases.

## 3. Usefulness and Problems of Direct NFAT Regulation

According to the progress in elucidating the role of NFAT in various diseases, the potential application of NFAT-targeting therapies has been expanded. Cyclosporine and/or tacrolimus, initially developed as immunosuppressive agents for organ transplantation, are currently used for many allergic and autoimmune diseases, such as atopic dermatitis, bronchial asthma, RA, ulcerative colitis, lupus nephritis, myasthenia gravis, Behcet’s disease, regenerative anemia, psoriasis, nephrotic syndrome, and Kawasaki disease. It has been reported that the incidence of dementia in patients who received cyclosporine or tacrolimus after transplantation was significantly lower than that in the general population [65]. However, serious and wide-ranging side effects, including increased risk of infectious diseases due to immunosuppression; organ disorders, such as those of the kidney, liver, and pancreas; cardiovascular disorders, including hypertension; nervous system disorders; and malignancies are major problems in using these drugs [66,67,68]. Their prolonged application further exacerbates side effects, thereby deteriorating the quality of life (QOL) of patients.

Numerous side effects of cyclosporine and tacrolimus are caused, at least in part, by the fact that they do not act directly on NFAT, but on CN, which catalyzes the dephosphorylation of NFATcs. Cyclosporine and tacrolimus form a complex with distinct intracellular proteins, cyclophilin and FK-binding proteins, respectively. These drug–protein complexes bind to similar sites in CN and inhibit phosphatase activity [69]. Although the possible treatment of breast cancer via CN-mediated cyclin D1 dephosphorylation has also been suggested [70], more than 50 substrate molecules other than NFATcs are regulated by CN [71,72,73]. Due to the widespread function of CN, the strategy for NFAT regulation by targeting CN activity may not lead to an improvement in patient QOL.

Therefore, to address the concerns regarding the side effects of CN inhibition, new agents that directly regulate NFATcs activity have been developed. NFATcs have been shown to interact with CN via two CN binding regions (CNBRs) located in the CRD. Based on the common sequence of the *N*-terminal side CNBR (CNBR1) of NFATcs, a modified 16 amino acid peptide (MAGPHPVIVITGPHEE) that exhibited enhanced affinity for CN was developed. The peptide, named VIVIT, based on its core sequence [74], suppressed the activation of NFATcs by competing with the conjugation of NFATcs to CN. The existing immunosuppressant-like pharmacological effects without expected side effects were obtained by the VIVIT peptide. Thus, the prevention of allograft rejection in islet-transplanted mice by tacrolimus was associated with a dose-dependent decrease in insulin secretion. On the other hand, essentially the same inhibitory effect on allograft rejection was achieved in mice administered the polyarginine-conjugated VIVIT peptide (11R-VIVIT) without impacting insulin secretion [75]. Introduction of the VIVIT peptide using an adenovirus-associated vector into AD model mice ameliorated the morphological neurodegenerative changes around amyloid β plaques [51]. Furthermore, the suppressive effect of the VIVIT peptide has been indicated in mouse models of heart disease, colitis, bronchial asthma, and type 2 diabetes [76,77,78,79]. Potential applications of the other types of direct NFAT inhibitors, such as A-285222 and INCA-6, to type 2 diabetes, diabetic retinopathy, and age-related macular degeneration, have also been demonstrated [80,81] (Table 1).

## 4. Significance of Selective Control of Specific NFAT Isoforms

Molecules that directly regulate NFATcs, such as VIVIT, may be useful for treating multiple diseases, although their clinical applications have not yet been achieved. Although the fewer side effects of the direct-acting NFAT inhibitors compared to cyclosporine or tacrolimus are promising, whether a more potent pharmacological action is expected in the direct inhibitors is unclear. Since the phenotypes of genetically modified mice for each NFAT are divergent, as mentioned above, outputs in the transcription of various target genes by regulating each NFAT isoform are expected to be different. The expression of IL-4, a typical T cell cytokine, is strongly suppressed in NFATc1-deficient T cells [82,83] but enhanced in some NFATc2-deficient mice [22,84,85]. For controlling IL-4 production by T cells, selective inhibition of NFATc1 alone may be more effective than targeting the entire NFAT family. However, by utilizing genetically modified mice systems alone, it is difficult to elucidate the detailed contribution of each NFAT isoform to the transcription of individual genes.

The potency of NFATc1 and NFATc2 to activate the transcription of cytokine genes in T cells and the corresponding mechanisms were comparatively analyzed by their ectopic introduction into T cells with careful adjustment of the expression levels. Both NFATc1 and NFATc2 augmented stimulation-induced IL-2 and GM-CSF expression. However, for TNF-α and IL-13 transcription, NFATc2 strongly enhanced transcription, whereas NFATc1 showed little contribution [86]. Conversely, IL-4 expression was more strongly induced by NFATc1. The difference in the potency of these isoforms to activate the transcription of cytokines might be linked to the different phenotypes observed in the corresponding knockout mice, as described above, typically in the case of IL-4. Moreover, by employing T cells introduced with various chimeric NFAT molecules in which each functional domain was exchanged between NFATc1 and NFATc2, it was clarified that the functional difference between these isoforms is caused, at least in part, by the deficiency of the *C*-terminal transcriptional activation domain in a dominant NFATc1 variant [86].

NFAT collaborates with other transcription factors to exhibit its transcription activation property in many genes [12,13,14]. In particular, on the IL-2 and TNF-α promoters, NFAT has been shown to associate with different co-effector heterodimer molecules, such as Jun/Fos (activator protein 1) and Jun/activating transcription factor 2, respectively [12,14,87]. NFATc1 and NFATc2 may exert different regulatory functions on the same cytokine gene by distinctively interacting with their individual co-effectors based on the different domain structures [86].

**Figure 2:**
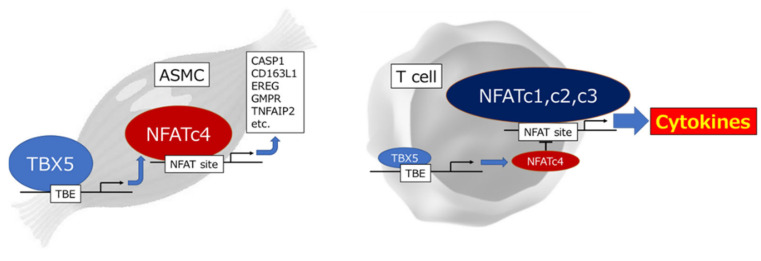
The different functional role of NFATc4 in aortic smooth muscle cells (ASMCs) and T cells through T-box factor 5 (TBX5)-mediated transcriptional regulation. CASP1; caspase 1, CD163L1; CD163 molecule like 1, EREG; epiregulin, GMPR; guanosine monophosphate reductase, TBE; T-box factor binding element, TNFAIP2; tumor necrosis factor-alpha-induced protein 2.

The differences in the tissue expression patterns of NFATcs have been investigated. In T cells, where NFAT plays an important role in their function, the NFATc4 level is much lower than that of other NFATcs [15]. Remarkably, cytokine production induced in human T cells upon activation was strongly suppressed by ectopic expression of NFATc4 [88]. The difference in the role of each NFAT isoform in the regulation of IL-2 expression was further examined by knockdown of endogenous NFATcs in Jurkat cells, a human T cell line that expresses NFATc4 at a relatively high level, by introducing the corresponding small interfering RNA. Stimulation-induced IL-2 expression was diminished by NFATc1, NFATc2, or NFATc3 knockdown but enhanced by NFATc4 knockdown, suggesting that NFATc4, which is slightly expressed in T cells, plays a suppressive role in cytokine expression. T cells might have evolved to acquire their characteristic features, the cytokine-producing ability, by reducing the expression of the suppressive NFAT isoform, NFATc4. On the other hand, NFATc4 is highly expressed in aortic smooth muscle cells (ASMCs) and regulates the expression of genes involved in their development and differentiation. The difference in the expression and resulting distinct function of NFATc4 in T cells and ASMC were regulated by the expression level of T-box transcription factor TBX5 (Figure 2) [88].

In addition to Ca^2+^-independent regulation of NFAT5, differences in the regulatory mechanisms of NFATcs have been reported. Kar et al. demonstrated that NFATc2 was activated by Ca^2+^ microdomains near open store-operated Ca^2+^ release-activated Ca^2+^ channels, whereas NFATc3 activation further required nuclear Ca^2+^ mobilization [89]. It seems that NFAT isoforms play individual complex roles through diversity in expressed tissues, functions, and regulatory mechanisms.

## 5. New Intramolecular Regions Involved in CN/NFAT Interactions

The identification of the opposite function among NFAT isoforms has improved the significance of selective means to control specific NFAT isoforms. Thus, NFAT isoform inhibitors may be superior to cyclosporine and tacrolimus not only in reducing side effects but also in augmenting the pharmacological efficacy. Therefore, the molecular mechanisms that enable isoform-selective control of NFAT have been investigated. In our recent study, detailed CN-binding properties were compared among CNBR1, CNBR2, and their intermediate region proteins, as well as the entire CRD of NFATcs, by employing recombinant proteins expressed in *Escherichia coli* (*E. coli*) (Figure 1). The catalytic subunit of CN, CNA, was also expressed and purified, and the binding affinity of each NFAT region with CNA was quantitatively assessed. However, despite employing several techniques for evaluating protein–protein interactions, such as the biophysical interaction analysis method using surface plasmon resonance (such as Biacore) and amplified emission proximity homogeneous assay technology, we could not obtain reasonable findings. Recombinant proteins normally contain impurities, and their purity depends on not only the size, sequence, and properties of their amino acids but also the expression and purification methods. Since these sophisticated techniques we initially applied are designated for analyzing interactions between highly purified molecules, they might not distinguish the target proteins and their impurities. To circumvent this distress, a new quantitative immunoprecipitation method was developed by employing highly purified proteins obtained by utilizing the DYKDDDDK tag inserted at the *C*-terminal end, in which only the full-length proteins expressed in *E. coli* were theoretically included. Owing to these technical improvements, the binding affinity between each NFAT region and CNA was successfully compared (Table 2).

**Figure 3:**
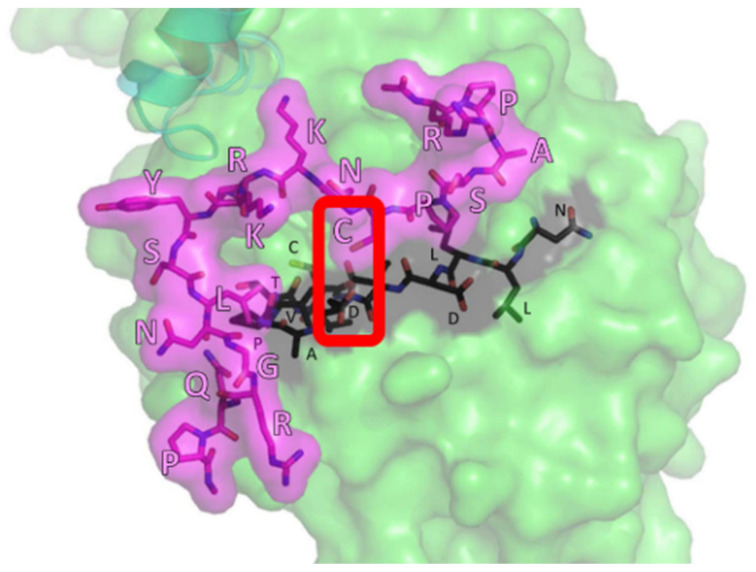
Putative three-dimensional structure of calcineurin A (CNA; green) and NFATc1 (pink) complex. The amino acids essential for the complex formation between NFATc1 (Cys263) and CNA (Asp82) are indicated by the red frame.

Similar CNA-binding activity with CRD was observed for all NFATcs. The CNBR1 region contributed to the interaction with CNA in all NFATcs, albeit with some differences in affinity. The CNBR2 region of NFATc1, NFATc3, and NFATc4 participated in CNA binding almost equally, while the binding affinity between NFATc2-CNBR2 and CNA was low, about 1/10th of that of other NFATcs. These results were in close agreement with previous findings obtained from qualitative experiments [90,91]. Remarkably, we found a novel CNA binding region in CRD that might exhibit the isoform-selective function. The intermediate region between CNBR1 and CNBR2 of NFATc1 and NFATc4, but not the other NFATcs, showed significantly strong binding activity to CNA (Table 2). Ectopic expression of this region, named CNBR3, in BHK cells that constitutively expressed fluorescence-labeled CRD proteins suppressed stimulation-induced nuclear translocation of NFATc1-CRD but not NFATc2-CRD. The interaction between NFATc1-CNBR3 and CNA was not affected by the VIVIT peptide or another CNA binding sequence peptide (DSSGDQFLSVPSPFTW) derived from the CNBR2 sequence, suggesting that the CNBR3 binding region in CNA is different from the regions responsible for interactions with CNBR1 and CNBR2. The binding regions of CNBR3 and CNA were narrowed down by competition assay using NFATc1-derived partial peptides and mass spectrometry with photoaffinity technology. Eighteen amino acids in NFATc1 (Arg258 to Pro275) and 13 amino acids in CNA (Asn77 to Gly89) were identified as the region involved in this binding (Figure 3) [92]. Amino acid substitution experiments, based on the binding model derived from the molecular operating environment (MOE) integrated computational system, further revealed that the interaction between Cys263 in NFATc1 and Asp82 in CNA was particularly essential for their binding (Figure 3).

## 6. Prospects for Disease Treatments Targeting the New Binding Region

**Figure 4:**
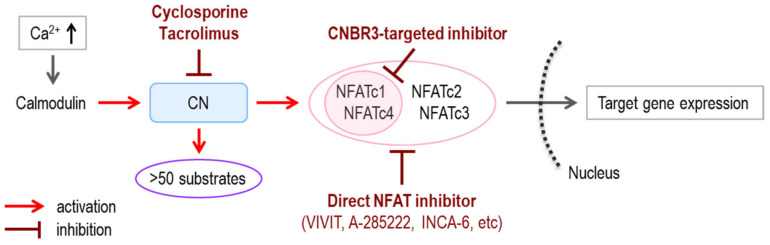
Schematic points of action of selective and non-selective NFAT inhibitors. CN; calcineurin, CNBR3; CN-binding region 3.

The potency and quality of novel means to control NFAT activity by targeting CNBR3, which probably affects only NFATc1 and NFATc4, probably differ from those of cyclosporine and tacrolimus. The stronger pharmacological effects of these immunosuppressants are expected in the new approach, especially for treating diseases where functional incompatibility occurs among NFAT isoforms. As described above, the relatively potent involvement of NFATc1 has been suggested in several diseases such as cancer, osteoporosis, and allergic disorders, while the selective role of NFATc4 has been suggested in AD. The effectiveness of immunosuppressants and/or direct NFAT inhibitors on those diseases was experimentally proven, although their clinical usages have not been approved. Therefore, the application of isoform-selective NFAT control methods promisingly achieved by targeting CNBR3 seems to be more appropriate and feasible at least to treat those diseases (Figure 4, Table 3). Since it was recently reported that cyclosporine and tacrolimus reduce sperm motility [93], the development of isoform-selective NFAT inhibitors that can be distinguished from classical immunosuppressants may be helpful as a countermeasure against the declining birth rate, which is becoming a serious problem in some countries.

## 7. Conclusions

The NFAT family of transcription factors with diverse functions is important not only in the immune system but also in various biological events. The findings of many studies to date indicate the overall picture of the physiological role of the NFAT family members and their concrete functions associated with several diseases. NFAT isoforms exhibit vast diversity because each isoform functions in an overlapping or opposite manner with respect to expression patterns within cells and tissues as well as to the functional characteristics, including participation in various diseases. Therefore, selective control of the individually contributing NFAT isoforms may be a key strategy for treating diseases, particularly those involving the corresponding isoforms. Our latest results show that pyrogallol derived from Awa tea associates with CNBR3 and consequently inhibits NFATc1 activity and IL-9 gene expression (manuscript in preparation). Even half a century after the discovery of cyclosporine, there are still many issues restricting the clinical application of therapeutic methods based on the direct control of NFAT. It is further complicated by recent investigations indicating the new regulatory mechanism of NFAT activity through SUMOylation [94] and the regulatory T cell induction dependent on the threshold value of total NFAT members [95]. However, the identification of new CN-binding regions that selectively contribute to specific NFAT-isoforms may lead to the development of innovative therapeutic strategies for improving the QOL of many patients suffering from refractory diseases or diseases for which there is no cure.

## Figures and Tables

**Table 1:** Functions and diseases associated with NFAT family members.

Isoform	Regulation Intracellular Localization	Involvement in Organ/Tissue Formation or Cell Function	Abnormal NFAT Activity	
			Human	Knockout Mouse
NFATc1	Calcineurin Cytoplasm (in resting cells) Nucleus (in activated cells)	Heart valve/septum formation [5,17], angiogenesis [32,33], osteoclast formation [28], T cell proliferation/Th2 differentiation [82,83]	Pancreatic cancer [31], colon cancer (increased risk of metastasis) [36], leukemia [35,37], lymphoma [39]	Embryonic lethality (circulatory failure) [5]
NFATc2		Cell cycle arrest/cell growth inhibition (normal cells) [26,41], activation-induced cell death [59], regulation of angiogenesis [45]	Pancreatic cancer [44], lung adenocarcinoma (> stage II) [43], mild cognitive impairment [50]	Increased immune reactivity [21,22], allergic contact hypersensitivity [59], lymphoma [42], lymphocyte hyperplasia (NFATc2/c3 DKO) [53],
NFATc3		-	Lung squamous cell carcinoma (well-differentiated cancer) [43], glioma [46]	lymphocyte hyperplasia (NFATc2/c3 DKO) [53], embryonic lethality (vascular hypoplasia, NFATc3/c4 DKO) [18]
NFATc4		Nervous system assembly/spatial memory formation [6,23]	Skin cancer [47], severe cognitive impairment [50], Alzheimer’s disease [50]	Spatial memory dysplasia [23], embryonic lethality (vascular hypoplasia, NFATc3/c4 DKO) [18]
NFAT5	mainly osmotic pressure cytoplasm and nucleus	Cell protection under hypertonic stress [8], homeostasis [9]	Rheumatoid arthritis [63]	Mostly embryonic lethality (decreased cardiac function, renal atrophy) [19,20]

DKO: double knock-out.

**Table 2:** Contribution of calcineurin (CN) binding regions (CNBRs) to CN/NFAT interaction.

Domain/Region	NFATc1	NFATc2	NFATc3	NFATc4
CRD	+++	+++	+++	+++
CNBR1	++	+++	++	++
CNBR2	++	+	++	++
CNBR3	++	−	−	++ ^1^

^1^ Affinity to CNA (*K*_d_, μM): <0.1; +++, 0.1 < 1; ++, 1 < 10; +, 10<; −. CRD: calcium regulatory domain.

**Table 3:** Current and potential application of NFAT inhibitors.

Application	Cyclosporin Tacrolimus	Direct NFAT Inhibitor	CNBR3-Targeted Inhibitor
Transplantation	○	△	
Atopic dermatitis	○		■
Bronchial asthma	○	△	■
Rheumatoid arthritis	○		
Ulcerative colitis	○	△	
Lupus nephritis	○		
Myasthenia gravis	○		
Behcet’s disease	○		
Regenerative anemia	○		
Psoriasis	○		
Nephrotic syndrome	○		
Kawasaki disease	○		
Alzheimer’s disease	△	△	■
Cardiac hypertrophy		△	
Type2 diabetes		△	
Osteoporosis	△	△	■
Cancer	△		■
Retinopathy	△	△	■
Existing and potential side effects	Sever	Moderate	Mild

○: clinically approved, △: experimentally proven, ■: suggested.

## Supplementary Materials

The following are available online at https://www.mdpi.com/1422-0067/22/5/2725/s1.
