# Supplementary material for: Isoform-Selective NFAT Inhibitor: Potential Usefulness and Development
Source: Int J Mol Sci. 2021 Mar 8;22(5):2725. doi: 10.3390/ijms22052725 (PMC7962815; doi:10.3390/ijms22052725)
Supplement: Supplementary file 1 [file ijms-22-02725-s001.pdf]

Supplementary files

Isoform-Selective NFAT Inhibitor: Potential Usefulness and Development

Noriko Kitamura [1], and Osamu Kaminuma [1,2,*]

[1] Laboratory of Allergy and Immunology, The Tokyo Metropolitan Institute of Medical Science, Tokyo 156-8506, Japan; kitamura-nr@igakuken.or.jp, okami@hiroshima-u.ac.jp
[2] Department of Disease Model, Research Institute of Radiation Biology and Medicine, Hiroshima University, Hiroshima 734-8553, Japan; okami@hiroshima-u.ac.jp
* Correspondence: okami@hiroshima-u.ac.jp; Tel.: +81-82-257-5819

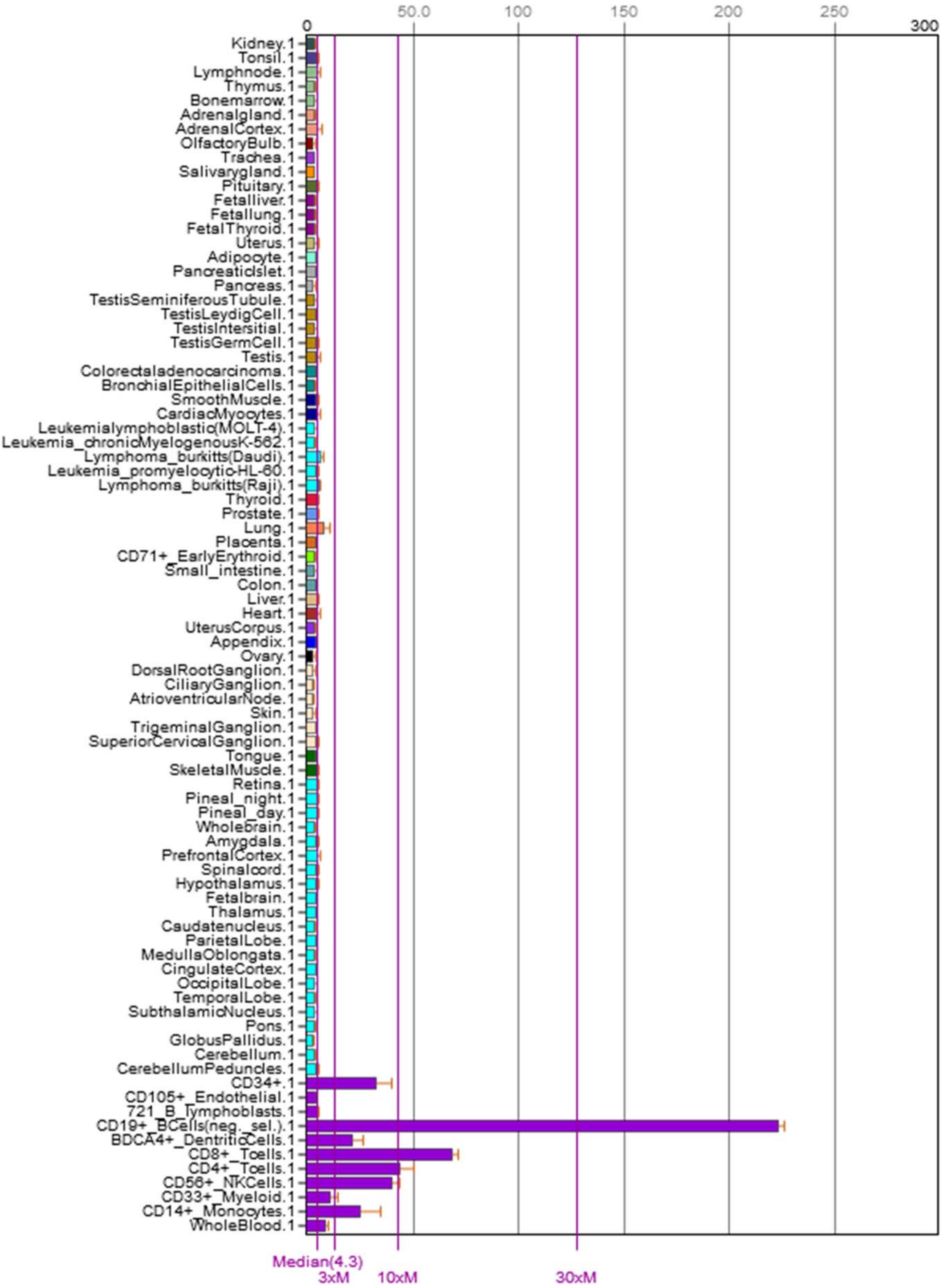

Figure S1. Expression pattern of NFATc1. The mRNA expression pattern of human NFATc1 was searched by BioGPS (http://biogps.org/).

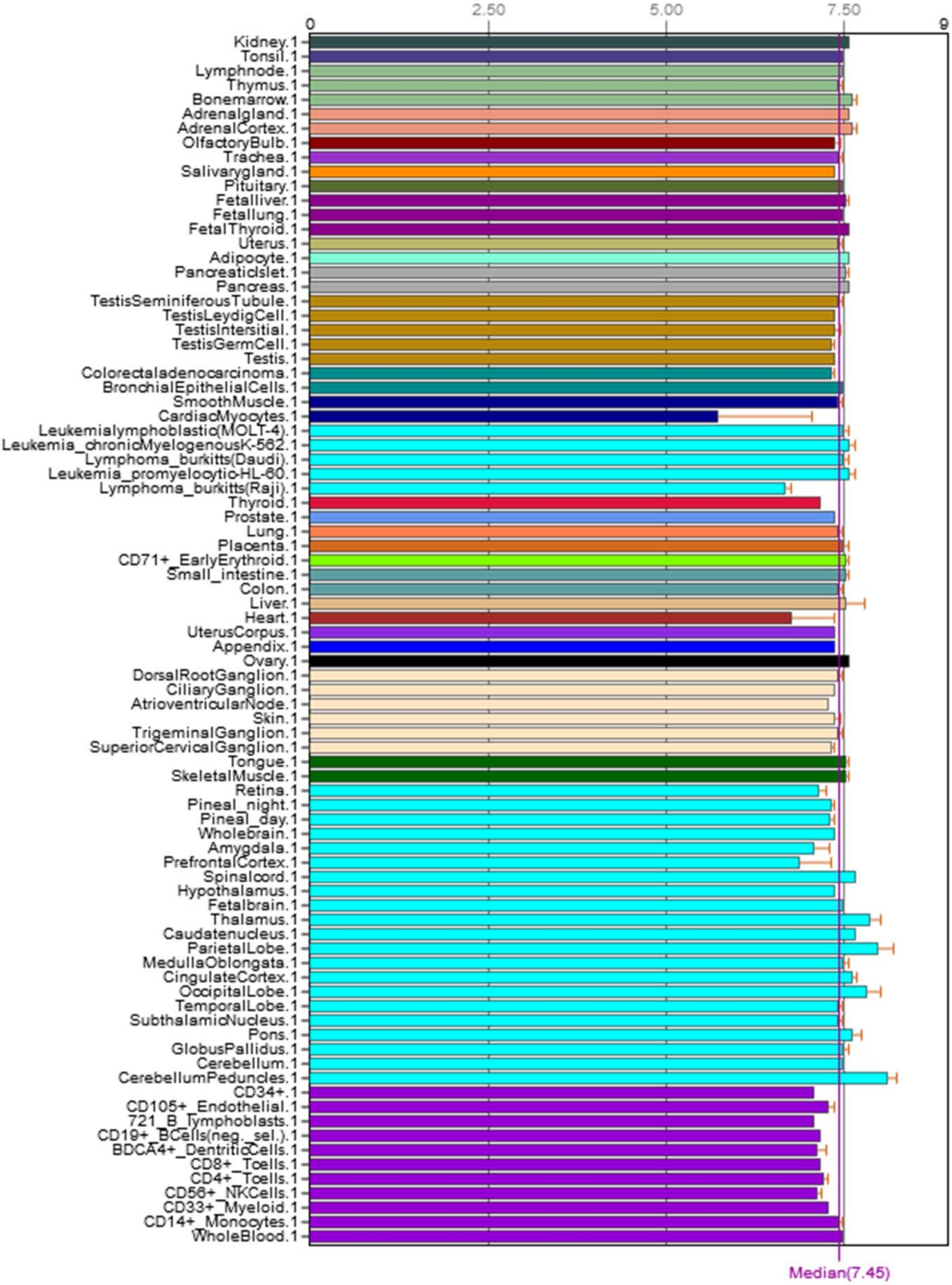

Figure S2. Expression pattern of NFATc2. The mRNA expression pattern of human NFATc2 was searched by BioGPS (http://biogps.org/).

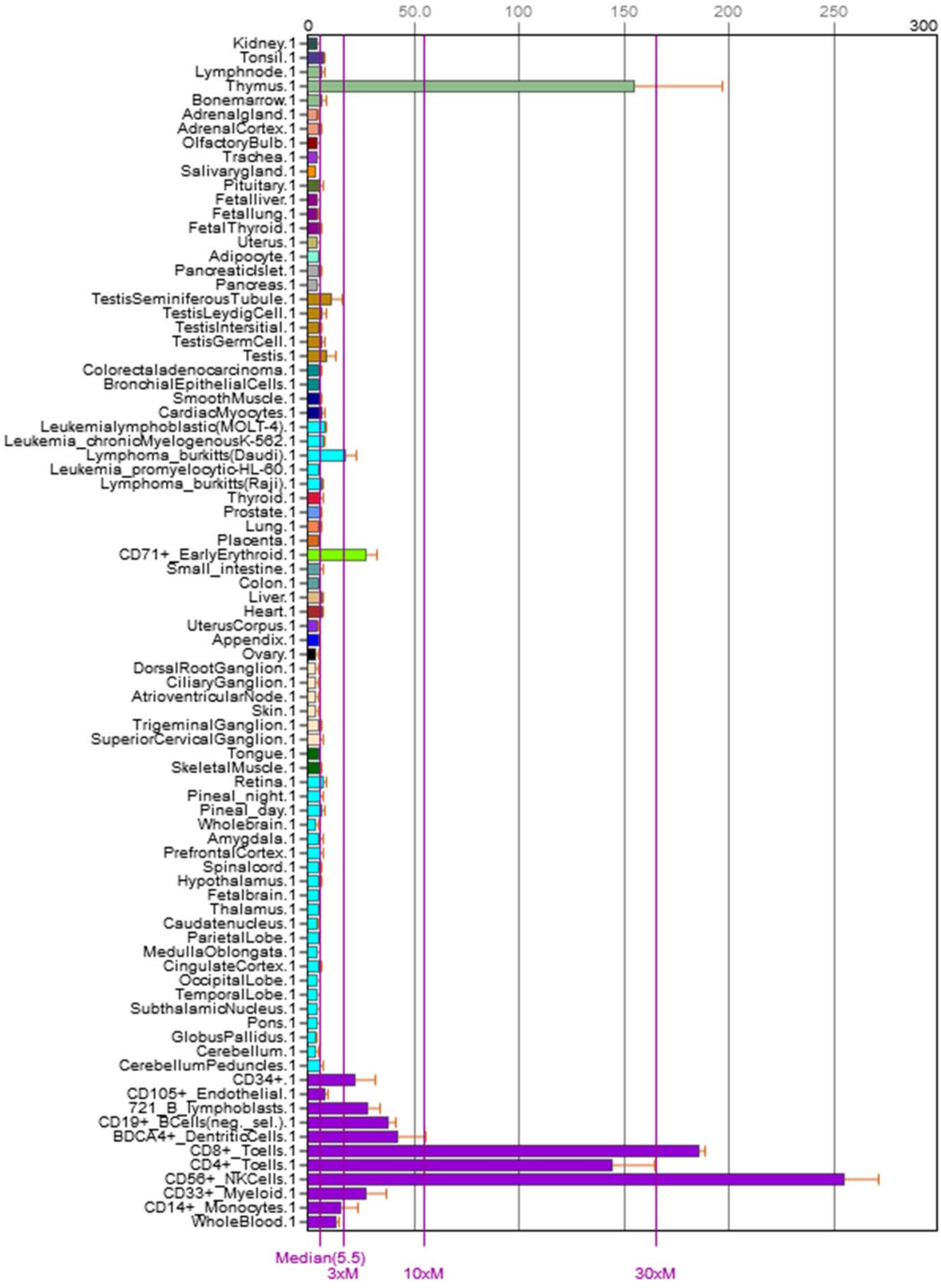

Figure S3. Expression pattern of NFATc3. The mRNA expression pattern of human NFATc3 was searched by BioGPS (http://biogps.org/).

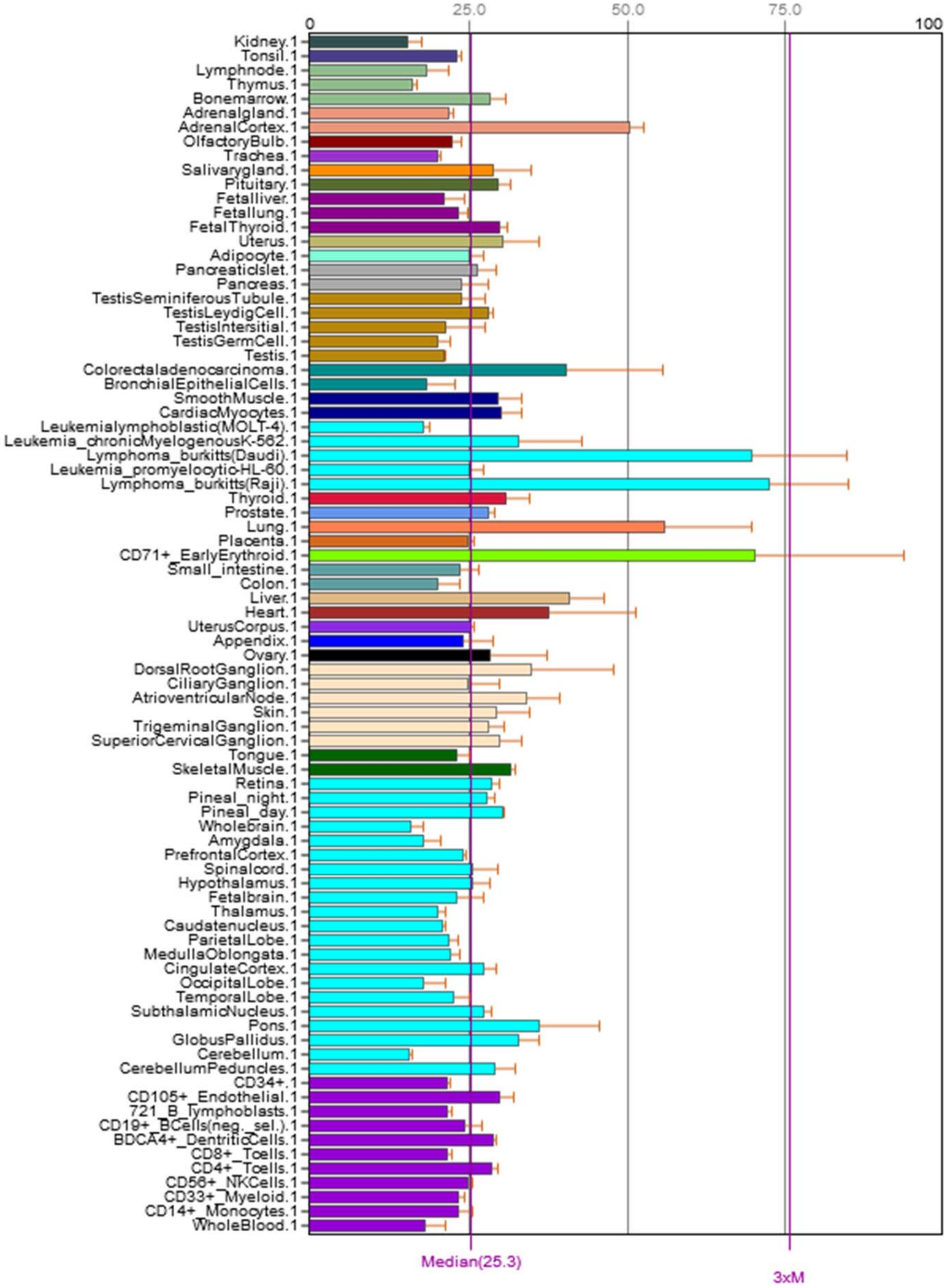

Figure S4. Expression pattern of NFATc4. The mRNA expression pattern of human NFATc4 was searched by BioGPS (http://biogps.org/).

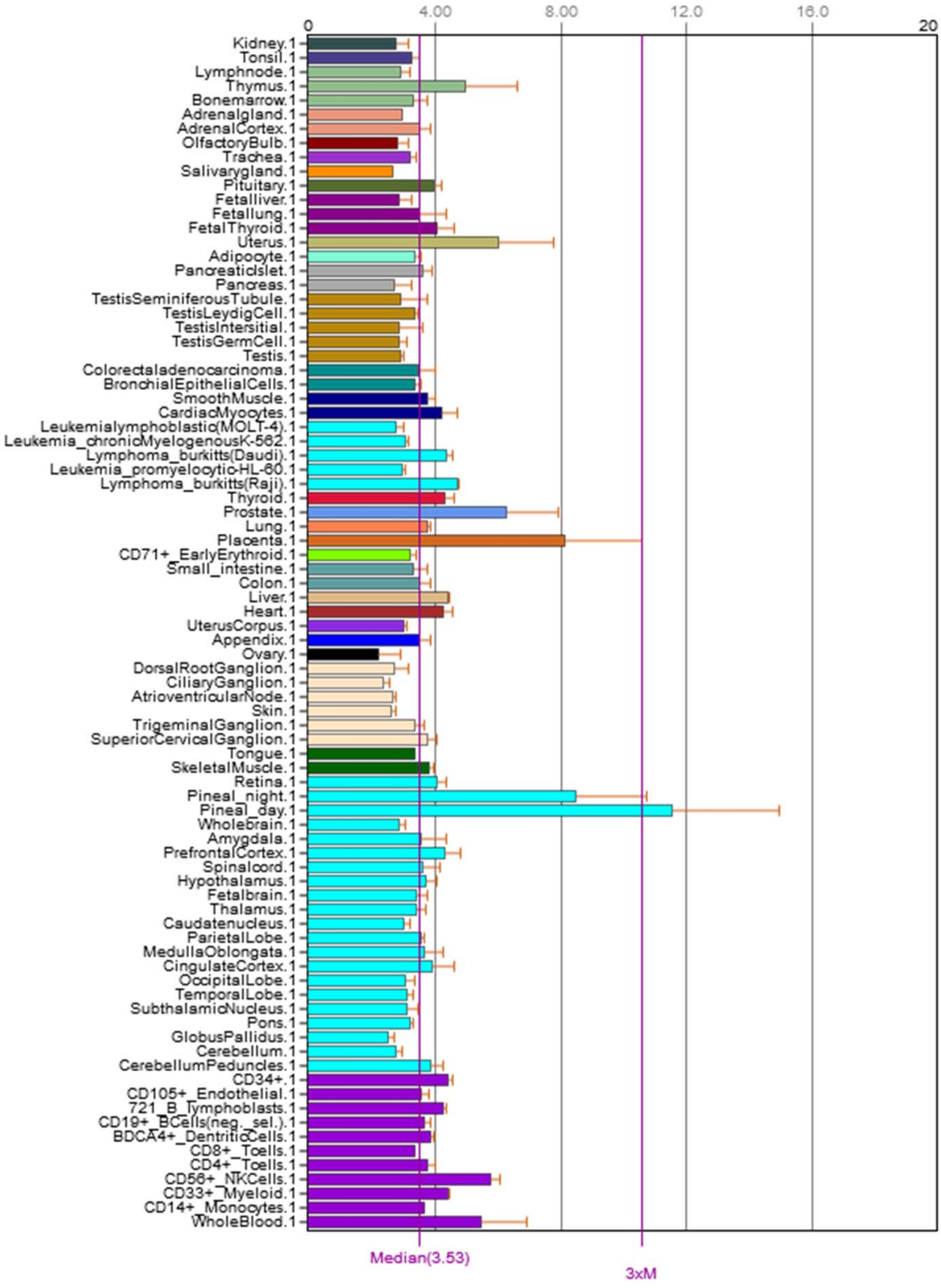

Figure S5. Expression pattern of NFAT5. The mRNA expression pattern of human NFAT5 was searched by BioGPS (http://biogps.org/).
